# Assembly and Trafficking of Homomeric and Heteromeric Kainate Receptors with Impaired Ligand Binding Sites

**DOI:** 10.1007/s11064-018-2654-0

**Published:** 2018-10-09

**Authors:** Caroline L. Scholefield, Palmi T. Atlason, David E. Jane, Elek Molnár

**Affiliations:** Centre for Synaptic Plasticity, School of Physiology, Pharmacology and Neuroscience, University of Bristol, Biomedical Sciences Building, University Walk, Bristol, BS8 1TD UK

**Keywords:** Kainate receptors, Glutamate, Ligand binding, Site-directed mutagenesis, Subunit assembly, Trafficking, Ca^2+^ imaging

## Abstract

Kainate receptors (KARs) are a subfamily of ionotropic glutamate receptors (iGluRs) mediating excitatory synaptic transmission. Cell surface expressed KARs modulate the excitability of neuronal networks. The transfer of iGluRs from the endoplasmic reticulum (ER) to the cell surface requires occupation of the agonist binding sites. Here we used molecular modelling to produce a range of ligand binding domain (LBD) point mutants of GluK1–3 KAR subunits with and without altered agonist efficacy to further investigate the role of glutamate binding in surface trafficking and activation of homomeric and heteromeric KARs using endoglycosidase digestion, cell surface biotinylation and imaging of changes in intracellular Ca^2+^ concentration [Ca^2+^]_i_. Mutations of conserved amino acid residues in the LBD that disrupt agonist binding to GluK1–3 (GluK1-T675V, GluK2-A487L, GluK2-T659V and GluK3-T661V) reduced both the total expression levels and cell surface delivery of all of these mutant subunits compared to the corresponding wild type in transiently transfected human embryonic kidney 293 (HEK293) cells. In contrast, the exchange of non-conserved residues in the LBD that convert antagonist selectivity of GluK1–3 (GluK1-T503A, GluK2-A487T, GluK3-T489A, GluK1-N705S/S706N, GluK2-S689N/N690S, GluK3-N691S) did not alter the biosynthesis and trafficking of subunit proteins. Co-assembly of mutant GluK2 with an impaired LBD and wild type GluK5 subunits enables the cell surface expression of both subunits. However, [Ca^2+^]_i_ imaging indicates that the occupancy of both GluK2 and GluK5 LBDs is required for the full activation of GluK2/GluK5 heteromeric KAR channels.

## Introduction

Kainate receptors (KARs) are members of the ionotropic glutamate receptor (iGluR) family [1, 2]. Presynaptically KARs modulate neurotransmitter (glutamate, GABA) release, postsynaptically they contribute to the slow component of excitatory postsynaptic current (EPSC), thereby influencing neuronal excitability and network dynamics [1, 3]. In addition to ionotropic activities, KARs exert their effects through metabotropic signalling pathways [4]. There are five KAR subunits: GluK1–5 (previously GluR5–7, KA-1 and KA-2) with different functional roles. Each subunit contributes to the formation of the KAR channel pore and contains a unique ligand binding domain (LBD) [5] with distinctive pharmacological properties [6]. The majority of native KARs are tetramers, made up of heteromeric combinations of GluK1–5 subunits [7]. Glutamate binding to the LBD triggers the opening of the KAR ion channel, which is permeable to Na^+^ and K^+^ [6]. Q/R editing in the channel pore forming domains of GluK1 and GluK2 determines the Ca^2+^ permeability of KARs [8]. KAR localisation at the cell surface is regulated by trafficking motifs for endoplasmic reticulum (ER) retention and ER export, alternative splicing, editing, associated proteins, post-translational modifications and actions of pharmacological and molecular chaperones [9, 10]. GluK1–3 traffic to the cell surface in both homo and heteromeric forms. However, GluK4 and GluK5 possess ER retention signals which are only alleviated in heteromeric assemblies with GluK1–3 [11–13]. Disruption of ligand binding to GluK2 leads to intracellular retention of subunits [14]. However, subunits with impaired LBD are able to assemble with other wild type (WT) KAR subunits with functional LBD, which enables their trafficking to the cell surface [14–16]. It has been proposed, that ligand binding in the ER is required for ER exit [17]. While functional activation of KARs may not be required, it is likely that glutamate binding induced conformational changes are necessary during biosynthesis and trafficking [14–17]. For example, the link between desensitization and ER exit has been made with KAR mutants, where engineered disulfide bridges across the ligand binding domain interface blocked GluK2 desensitization and retained mutant receptors in the ER [18].

Various KAR subunits have been implicated in different neurological and psychiatric disorders (e.g. temporal lobe epilepsy [19–21], autism [22, 23], mental retardation [24], migraine [25], schizophrenia [26], depression [27] and bipolar disorder [28]). The involvement of KARs in these pathological conditions and the possible underlying cellular and molecular mechanisms are discussed in recent reviews [3, 29–31]. Therefore, KAR subunit selective ligands could have therapeutic potential [6]. The availability of crystal structures of KARs [5, 6] and the identification of key amino acid residues that are responsible for specific pharmacological properties of subunits [32] created new opportunities for the production of novel ligands [33–35]. However, the development of selective and potent compounds and their functional characterisation require functional expression of recombinant KAR subunits in cell lines for electrophysiological analysis or fluorescence-based screening assays using fluorescent Ca^2+^ indicators [6, 36, 37]. Previous studies typically used homomeric GluK1, GluK2 or GluK3 KARs expressed in human embryonic kidney 293 (HEK293) cells to test the subunit selectivity and pharmacological properties of new ligands [6, 33]. Due to the ER retention of GluK4 and GluK5 homomers, it was not possible to test their specific pharmacological characteristics in cell-based functional assays. Furthermore, there are indications that the agonist and antagonist sensitivity of heteromeric receptors can differ from recombinant homomeric KARs [38]. Therefore, testing the pharmacological properties of various KAR subunit ligand binding sites in heteromeric assemblies would be advantageous. Due to the presence of different functional LBDs in a heteromeric KAR assembly, studying the pharmacological characteristics of a specific ligand biding site in isolation and testing the subunit selectivity of a drug is very challenging.

Because each KAR subunit possesses its own ligand binding site, we hypothesised that using site-directed mutagenesis to disable the ligand binding to one subunit in a heteromer would allow the pharmacological investigation of the intact LBD in another subunit. This approach would be particularly advantageous for the development of GluK4 and GluK5 selective ligands using cell-based assays, because these subunits do not form functional KARs on their own due to their ER retention [6, 11–13]. In this study we analysed changes in the biosynthesis, stability, cells surface expression and agonist-induced responses of homomeric and heteromeric KARs with various LBD mutations to examine the interplay between different ligand binding sites.

## Materials and Methods

### Analysis of the Ligand Binding Sites

The X-ray crystal structure of the rat GluK2 LBD in complex with kainate (Protein Data Bank identification code 1TT1 [39]) was used as a template for homology modelling of the GluK1 and GluK3 subunit ligand binding sites using SWISS-MODEL and the resultant models were examined using Accelrys DS Visualizer 3.1 (Accelrys, Inc. San Diego, CA) [32]. The residues in the GluK2 LBD that are important for agonist binding were mapped onto the LBDs of GluK1 and GluK3 using linear sequence alignment to determine homologous binding site residues within these subunits. Similar methodology was used starting from the X-ray crystal structure of the rat GluK1 LBD in complex with the GluK1 selective antagonist UBP310 to determine equivalent residues that would be involved in antagonist binding in the LBDs of GluK2 and GluK3 (see [32] for details). Residue numbering used excludes the signal peptide.

### Mutagenesis

The desired mutations of the human GluK1–2a(Q), GluK2a(Q) and GluK3a subunit encoding cDNAs were generated using the QuickChange II XL site directed mutagenesis kit (Stratagene, La Jolla, CA) following the protocol recommended by the manufacturer. Mutations were confirmed by full-length sequencing (Geneservice, Oxford, UK).

### Cell Culture and Transfection

HEK293 cells were grown in Dulbecco’s modified Eagles medium (DMEM, Sigma, Gillingham, UK) containing 10% (v/v) fetal bovine serum (Biosera, Uckfield, East Sussex, UK), 100 µg/ml penicillin, 100 µg/ml streptavidin and 2 mM l-glutamine (all from Invitrogen Ltd., Paisley, UK) at 37 °C in a humidified atmosphere of 5% CO_2_. For passaging, cells were washed three times in PBS (150 mM NaCl, 2.6 mM KCl, 10 mM Na_2_PO_4_, 1.9 mM KH_2_PO_4,_ pH 7.4), detached in trypsin and resuspended in DMEM for plating. Cells were transiently transfected using linear polyethylene as described previously [32] and harvested 24 h following transfection.

### Preparation of Membrane Fractions

Membrane fractions prepared from transiently transfected HEK293 cells were harvested in 10 mM NaHCO_3_ with complete protease inhibitor cocktail (Roche Diagnostics GmbH, Mannheim, Germany). Cells were lysed using an ultrasonic cell disruptor (2 × 10 s bursts at 10 W on ice; Microson, Qsonica, LLC., Newtown, CT, USA). Cell homogenates were centrifuged (1095×*g*, 20 min, 4 °C) to remove cellular debris. From the supernatants, membranes were pelleted at 40,000×*g* for 20 min at 4 °C. For radioligand binding assay, membranes were washed three times using repeated resuspension in 50 mM Tris (pH adjusted to 7.2 using citric acid) and centrifugation (40,000×*g*, 20 min, 4 °C). Protein concentrations were measured using a protein assay kit (Bio-Rad Laboratories, Hemel Hempstead, UK) before membrane fractions were snap frozen in liquid nitrogen and stored at − 80 °C.

### Radioligand Binding Assay

For radioligand binding assays using filtration with a Brandel cell harvester (model M-30; Brandel, Gaithersburg, MD, USA) [32], membranes were diluted in binding buffer (50 mM Tris buffered with citric acid, pH 7.2). Membrane proteins (150 µg) were incubated for 45 min with varying concentrations of [^3^H]kainate (37 MBq/ml; PerkinElmer Life and Analytical Sciences, Waltham, MA) or [^3^H]-(2*S*,4*R*)-4-methylgutamate ([^3^H]MG; 50.6 Ci/mmol; Tocris Cookson, Bristol, UK) on ice. Kainate (1 mM) or glutamate (1 mM) was added to parallel samples to determine nonspecific binding. Following rapid washing with binding buffer (3 × 4 ml), the radioactivity of filters was measured using a liquid scintillation counter (LS6500, Beckman High Wycombe, UK).

### Endoglycosidase Digestion

Membrane fractions prepared from transiently transfected HEK293 cells were solubilised in 1% Triton X-100 and 0.1% SDS in phosphate buffered saline (PBS; 150 mM NaCl, 2.6 mM KCl, 10 mM Na_2_PO_4_, 1.9 mM KH_2_PO_4,_ pH 7.4). Samples were centrifuged (21,255×*g*, 15 min, 4 °C) to remove debris. Endoglycosidase H (EndoH, 5 mU/100 µl of sample) or endoglycasidase F (EndoF; 1 U/100 µl of sample; Roche Diagnostics GMBH, Mannheim, Germany) were added to equal aliquots of solubilised membrane samples and incubated overnight at 37 °C [40] before immunoblot analysis.

### Cell Surface Biotinylation

Transfected HEK293 cells were washed three times with PBS and incubated for 30 min in EZ-link sulfo-NHS-*S*-*S*-biotin (0.5 mg/ml; Pierce Biotechnology, Rockford, IL, USA) as described previously [11, 41]. Membrane fractions prepared from cell surface biotinylated cultures were solubilised in lysis buffer (1% triton X100, 0.5% deoxycholic acid, 0.1% SDS, 1 mM EDTA, 100 mM NaCl, 50 mM Tris–HCl, pH 7.4 with complete protease inhibitor cocktail; Roche Diagnostics GmbH, Mannheim, Germany). Cellular lysates were centrifuged (21,255×*g*, 15 min, 4 °C) and 1/10th of the supernatant containing the solubilised membranes was used for acetone precipitation of proteins. To separate biotinylated cell surface and non-biotinylated intracellular proteins, the remaining fraction of lysate was incubated with a 1:1 mixture of streptavidin agarose beads (Sigma-Aldrich Company Ltd., Gillingham, Dorset, UK) for 3 h at 4 °C with mixing by rotation. Beads were washed in lysis buffer and 50 mM Tris–HCl (pH 7.5) before biotinylated cell surface proteins were eluted by heating (100 °C for 2 min) in SDS-polyacrylamide gel electrophoresis (SDS-PAGE) sample buffer (2% (w/v) SDS, 50 mM Tris–HCl (pH 6.8), 10% (v/v) 2-mercaptoethanol, 10% (v/v) glycerol and 0.1% (w/v) bromophenol blue) [11]. The β-actin content of biotinylated fractions was analysed on immunoblots to confirm that no intracellular proteins were labelled with biotin.

### Cycloheximide Treatment of Cells

Twenty-four hours after transfection, HEK293 cells were treated with the protein synthesis blocker cycloheximide (50 µg/ml; Sigma-Aldrich Company Ltd., Gillingham, Dorset, UK) to investigate potential differences in the degradation of wild type (WT) and mutant KARs subunits. Sister cultures were harvested at 0, 4, 8, 12, 24 and 36 h after the addition of cycloheximide and KAR subunit proteins were analysed in cell membrane lysates using immunoblotting as described previously [40].

### Immunoblot Analysis

Following solubilisation in SDS-PAGE sample buffer, equal aliquots of proteins (10–20 µg/lane) were separated on 8% (w/v) SDS-PAGE gels and electrophoretically transferred onto polyvinylidene difluoride membranes [11]. Blots were probed with 0.1–0.5 µg/ml previously validate immunoaffinity purified polyclonal rabbit antibodies to GluK1 [32], GluK2/3 [42], GluK5 [42, 43] (Millipore, Watford, UK) and mouse monoclonal anti-β-actin antibody (Abcam, Cambridge, UK). Previously described protocol was used to establish the linear sensitivity range of immunoreactivities for quantification using enzyme chemiluminescence (ECL) [44]. For the quantitative comparison of total KAR subunit levels, immunoreactivities were normalised to the β-actin content of each sample.

### Measurements of [Ca^2+^]_i_

Changes in intracellular Ca^2+^ concentration [Ca^2+^]_i_ were measured by following changes in Ca^2+^-sensitive dye (fura-2-AM; Sigma) fluorescence. Green fluorescent protein (GFP) co-expression was used to enable the identification of cells that express KAR subunits (~ 12%). Transfected HEK293 cells, that had been maintained for 24 h, were washed three times in HEPES buffered saline (HBS; 135 mM NaCl, 5 mM KCl, 10 mM HEPES, 1 mM MgCl_2_, 2 mM CaCl_2_, 30 mM glucose, pH 7.4) at 37 °C and loaded with 5 µM fura-2-AM in HBS for 45 min at 37 °C. Cells were than washed again in HBS three times and incubated for 15 min in the dark at room temperature (about 20 °C). Imaging was carried out using a continuous flow of HBS (2 ml/min at 20 °C) with a wash time of 5 min followed by addition of carbachol (10 µM) and kainate (25 µM; Tocris Bioscience, Bristol, UK) in the presence of the KAR desensitisation blocker concanavalin A (ConA; 0.3 mg/ml; Sigma [45]) for 5 min with a 10 min wash in between drugs. Cells were visualised through a x20 objective on a Nikon eclipse TE2000-S microscope and a Hamatsu ORCA-ER camera (Hamatsu Photonics, Welwyn Garden City, UK). For ratiometric measurements, the emission at 510 nm was measured at 5 s intervals during alternate excitation at 340 nm and 380 nm for 200 ms, by a Lamda DG-4 light source and wavelength switcher (Sutter Instrument, Novato, CA, USA). The ratio of intensity of the emission at 340 nm and 380 nm (340/380 ration) was calculated by the Volocity software (PerkinElmer, Coventry, UK) and used as a measure of [Ca^2+^]_i_. Matlab (Mathworks, Cambridge, UK) was used to calculate whether individual cells responded to kainate or carbachol. A positive response was recorded when 340/380 ratio was more than twice the standard deviation of the baseline measured immediately before drug application.

### Statistics

Statistical analysis was performed using an unpaired Student’s *t* test. The ‘*n*’ represents the number of independent experiments. For non-equal ‘*n*’ numbers, a two-sample equal variance *t* test was performed. A ‘*p*’ value of less than 0.05 was considered significant (*) and less than 0.02 was considered highly significant (**). Results are presented as mean ± SEM.

## Results

### Identification of Residues in GluK1–3 Subunits that are Involved in Ligand Binding

**Fig. 1:**
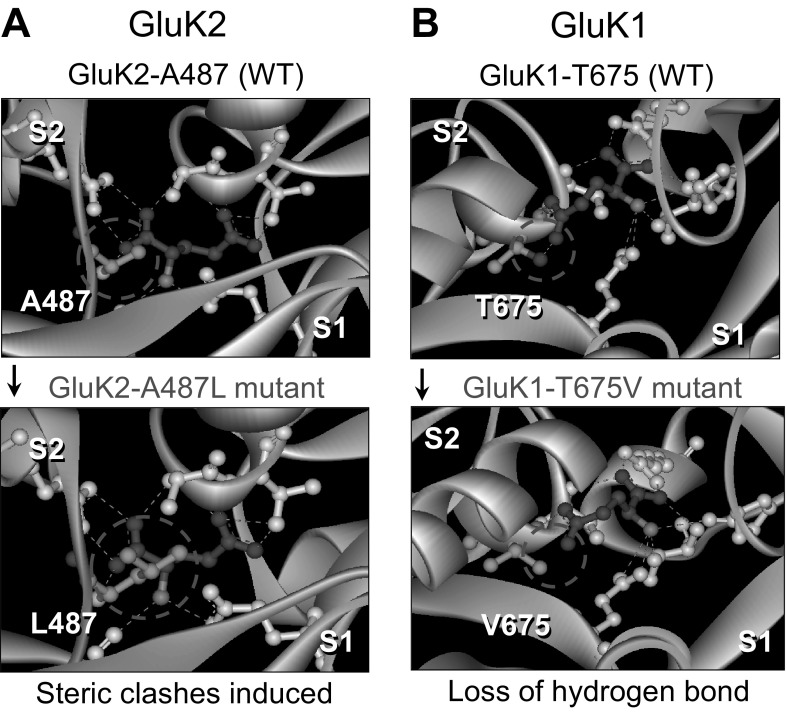
Identification of amino acid residues critical to agonist binding. Molecular modelling based on the crystal structures of the S1S2 ligand binding domains of GluK1 and GluK2 in complex with glutamate (purple) [39] was used to generate mutant subunits with impaired ligand binding sites. **a** It was predicted that glutamate binding can be prevented through the introduction of steric clashes by changing an Ala residue in the S1 domain of GluK2 (GluK2-A487) to a bulkier Leu (green molecule). **b** Modelling indicated that glutamate forms a hydrogen bond (green dotted line in red circle) with a Thr residue (green) in the S2 domain of GluK1 (GluK1-T675), which can be disrupted by mutating this residue to Val. (Color figure online)

To identify *conserved* residues within the ligand binding domains (LBDs) of GluK1–3 subunits that are critical to glutamate binding, high resolution crystal structures of the GluK1 and GluK2 S1S2 domains [39] were analysed (Fig. 1). These models predicted that conserved amino acid residues in the GluK2 S1 domain (GluK2-A487) and GluK1–3 S2 domains (GluK1-T675, GluK2-T659 and GluK3-T661) are important for agonist binding. Mutation of these residues would interfere with glutamate binding, by creating steric clashes (GluK2-A487L; Fig. 1a) or through the loss of a hydrogen bond (GluK1-T675V; Fig. 1b). Steric occlusion in the GluK2-A487L mutant is achieved by replacing the methyl group of GluK2-A487 with a bulkier isobutyl group via replacement with a leucine residue, which extends deeper into the binding cavity, thereby preventing agonist binding. The hydroxyl group of GluK1-T675 forms an important hydrogen bond with the distal carboxylate of agonists such as glutamate and kainate and its replacement with a methyl group in the GluK1-T675V mutant would therefore be expected to drastically reduce agonist affinity.

**Fig. 2:**
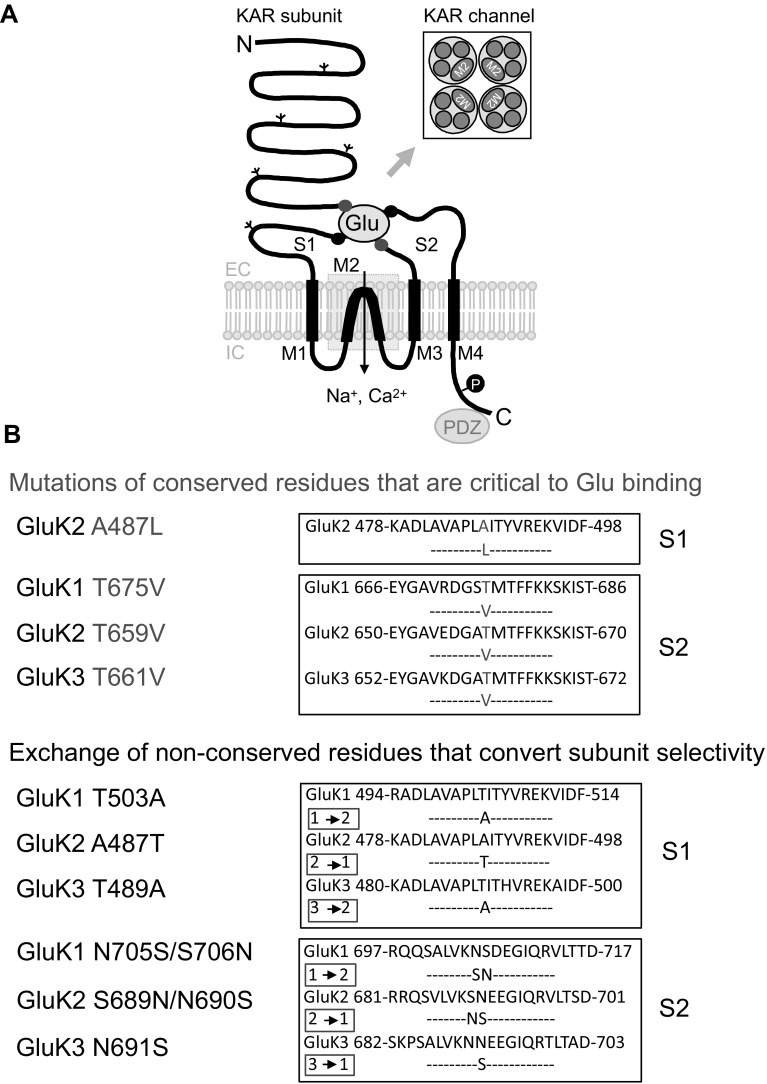
Mutation of key amino acid residues in the S1S2 ligand binding domains of GluK1–3. **a** Schematic model of kainate receptor (KAR) subunit with the S1S2 ligand binding sites and M1-4 membrane domains. *Glu* glutamate, *N* amino-terminus, *C* carboxy-terminus, *EC* extracellular, *IC* intracellular. Insert illustrates membrane domains in the KAR ion channel, which is formed by four subunits. **b** Mutations in the S1S2 ligand binding sites of GluK1–3 that disrupt glutamate (Glu) binding (red) or convert subunit selective characteristics of GluK1–3 without blocking Glu binding (blue; [32]). (Color figure online)

The identification and characterisation of *non-conserved* amino acid residues in the GluK1–3 S1 domains (GluK1-T503, GluK2-A487 and GluK3-T489) and S2 domains (GluK1-S706, GluK2-N690 and GluK3-N691) that determine antagonist subunit selectivity without blocking agonist binding to the receptor (Fig. 2) has been described in our previous study [32]. While the swapping of these non-conserved residues between GluK1–3 converts the antagonist selectivity of subunits, glutamate and kainate binding is retained [32]. Therefore, these mutants that convert subunit-selective characteristics of GluK1–3 LBDs (Fig. 2; [32]) together with wild type (WT) KAR subunits were used as controls in this study.

### Mutation-Induced Changes in [^3^H]kainate and [^3^H]MG Binding to GluK1–3

**Fig. 3:**
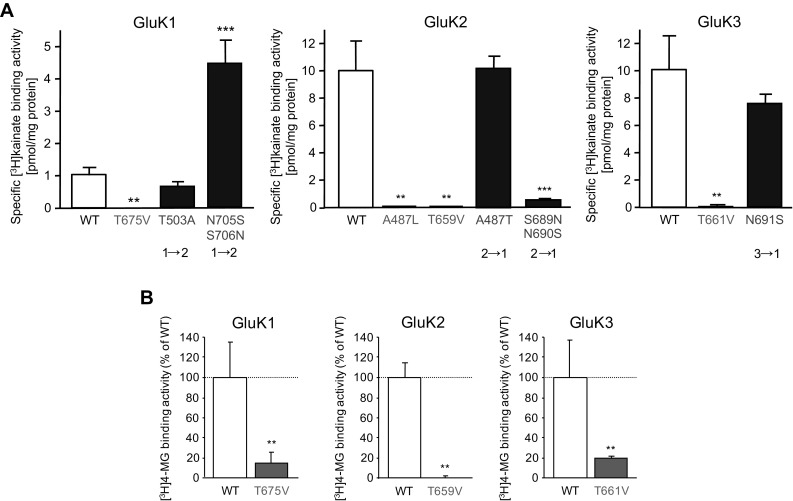
Modification of conserved amino acid residues within the GluK1–3 S1S2 domains disrupts agonist binding. HEK293 cells were transiently transfected with wild type (GluK1-WT, GluK2-WT, GluK3-WT) or LBD mutant (GluK1-T675V, GluK1-T503A, GluK1-N705S/S706N, GluK2-A487L, GluK2-T659V, GluK2-A487T, GluK2-S689N/N690S, GluK3-T661V, GluK3-N691S) GluK1–3 KAR subunit containing plasmids. After 48 h membranes were isolated and thoroughly washed by centrifugation and resuspension, before the radioligand binding assay was carried out. The presence of KAR subunits in membrane samples was confirmed using immunoblotting (not shown). Membrane fractions (150 µg protein/assay) were incubated with 100 nM [^3^H]kainate in the presence (non-specific binding) and the absence (total binding) of 1 mM kainate **a** or 10 nM [^3^H]-(2S,4R)-4-methylgutamate ([^3^H]MG) in the presence (non-specific binding) and the absence (total binding) of 1 mM glutamate (**b**). While the mutation of conserved residues (GluK1-T675V, GluK2-A487L, GluK2-T659V, GluK3-T661V) resulted a marked reduction in [^3^H]kainate (**a**) and [^3^H]MG (**b**) binding, exchanging residues between GluK1 and GluK2 at the T/A site of the S1 domain (GluK1-T503A, GluK2-A487T) produced no significant change in [^3^H]kainate binding. The exchange of N/S residues at the S2 domain increased [^3^H]kainate binding to GluK1-N705S/S706N and reduced the binding activity of GluK2-S689N/N690S and GluK3-N691S. LBD mutant GluK1–3 were compared to the corresponding WT subunit. Data are mean ± SEM (*n* = 3 independent determinations using three parallel samples in each experiment); **p < 0.01, ***p < 0.005, Student’s *t* test

To establish if the mutations that were introduced based on the crystal structure analysis had the desired effects on the agonist binding properties of mutant GluK1–3 KAR subunits (Fig. 2), radioligand binding assays were performed using [^3^H]kainate and [^3^H]MG (Fig. 3). Mutations of conserved residues in the GluK1–3 LBDs (GluK1-T675V, GluK2-A487L, GluK2-T659V and GluK3-T661V) significantly reduced both [^3^H]kainate (Fig. 3a) and [^3^H]MG (Fig. 3b) binding activities. In contrast, exchanging A/T non-conserved amino acid residues in GluK1 (GluK1-T503A) and GluK2 (GluK2-A487T) (Fig. 2b) produced no change in ligand binding activity compared to the corresponding WT subunits (Fig. 3a). The [^3^H]kainate binding activities of GluK1-N705S/S706N, GluK2-S689N/N690S and GluK3-N691S were shifted from the parent subunit towards that of the subunit the mutation was designed to imitate (Fig. 3a; [32]). These experiments confirmed that mutation of GluK1-T675, GluK2-A487, GluK2-T659 and GluK3-T661 disrupts agonist binding.

### Changes in the Expression Levels of Ligand Binding Site Mutant GluK1–3 Subunits

**Fig. 4:**
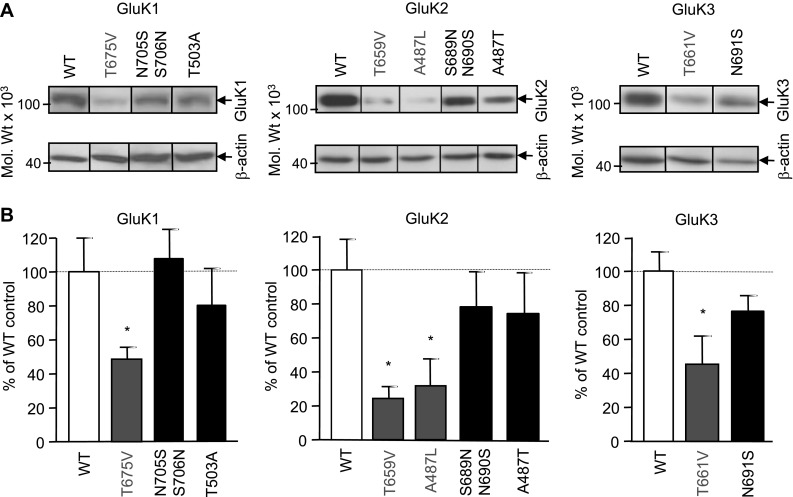
Mutations that disrupt agonist binding cause reduction in KAR subunit protein levels. **a** Membrane fractions prepared from transiently transfected HEK293 cells 24 h after transfection, were analysed using immunoblotting with previously validated GluK1 and GluK2/3 subunit selective antibodies. To reveal potential quantitative differences in total KAR subunit expression levels, samples were prepared from sister cultures transfected at the same time with the various WT and mutant subunit constructs and processed in parallel for immunoblotting. ECL exposure time was adjusted to the linear sensitivity range of immunoreactivities to allow quantitative comparisons between bands identified by the same antibody. **b** KAR subunit immunoreactivities were normalised to the β-actin content of the sample. Mutant GluK1–3 subunits with impaired LBDs (GluK1-T675V, GluK2-T659V, GluK2-A487L; red) show a significant decrease in subunit protein levels compare to the corresponding WT (GluK1-WT, GluK2-WT, GluK3-WT; white) or other LBD mutants that retained agonist binding activity (GluK1-N705S/S706N, GluK1-T503A, GluK2-S689N/N690S, GluK2-A487T, GluK3-N691S; blue). Data are mean ± SEM (*n* = 3–4), *p < 0.05, Student’s *t* test. (Color figure online)

Expression levels of WT and LBD mutant GluK1–3 subunit proteins (Fig. 2) were assessed 24 h after transient transfection of HEK293 cells using immunoblot analysis (Fig. 4a). Previously characterised GluK1 [32] and GluK2/3 [11, 32, 40, 42] specific antibodies were used for the identification of KAR subunits (Fig. 4a). While the expression levels of GluK1–3 subunits with functional LBDs (GluK1-N705S/706N, GluK1-T503A, GluK2-S689N/N690S, GluK2-A487T and GluK3-N691S) were similar to WT, there was a marked reduction in the level of subunits with impaired ligand binding (GluK1-T675V, GluK2-A487L, GluK3-T661V; Fig. 4b). Investigation of the degradation rates of different LBD mutant GluK1–3 subunits (Fig. 2) using cycloheximide treatment [40] indicate no significant differences at any of the investigated time points (4, 8, 12, 24 and 36 h following the addition of cycloheximide) compared to WT (not shown). These experiments indicate that there is a significant reduction in the level of GluK1–3 subunits with impaired ligand binding sites and this does not appear to be caused by increased degradation.

### Comparison of Intracellular Trafficking of GluK1–3 Containing Homomeric Wild-Type and Ligand Binding Site Mutant KARs

**Fig. 5:**
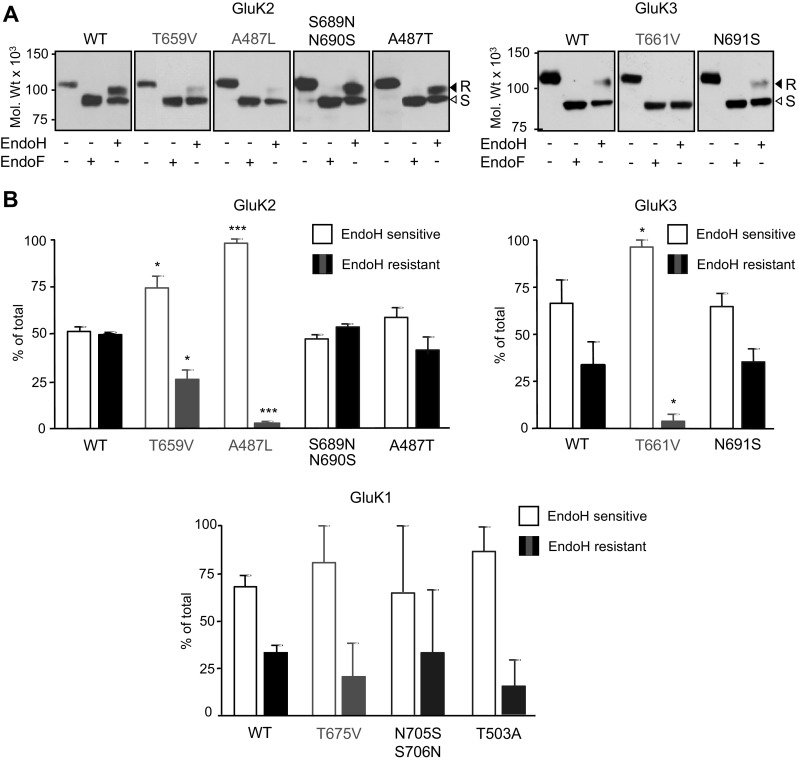
Homomeric KARs with impaired LBDs are retained in intracellular compartments. **a** Solubilised membrane fractions obtained from transiently transfected HEK293 cells expressing WT and mutant GluK2 and GluK3 subunits for 24 h were deglycosylated with EndoH or EndoF as indicated and analysed using immunoblotting with a rabbit GluK2/3 specific antibody. While EndoH resistant GluK2 and GluK3 were identified around 105 kDa (R, black arrow heads), the EndoH sensitive subunits showed a molecular weight reduction to ~ 95 kDa (S, white arrow heads). EndoF enzyme treatment of GluK2 and GluK3 produced a single band with molecular weight of ~ 93 kDa. Due to the identified differences in total expression levels of various KAR subunit mutants (Fig. 4), immunodetection of bands was optimised separately for the quantitative comparison of EndoH and EndoF sensitive/resistant bands for each construct. **b** Mutants with impaired LBDs (GluK2-T659V, GluK2-A487L and GluK3-T661V; red) show greater EndoH sensitivity than the corresponding WT subunits. In contrast, LBD conversion mutants of GluK2 and GluK3 that retain ligand binding (GluK2-S689N/N690S, GluK2-A487T, GluK3-N691S) show no significant change in EndoH sensitivity compared to WT. There were no detectable differences in the EndoH sensitivity of GluK1-WT, GluK1-T503A, GluK1-N705S/S706N and GluK1-T675V. Data are mean ± SEM (*n* = 3), *p < 0.05, ***p < 0.005, Student’s *t* test. (Color figure online)

**Fig. 6:**
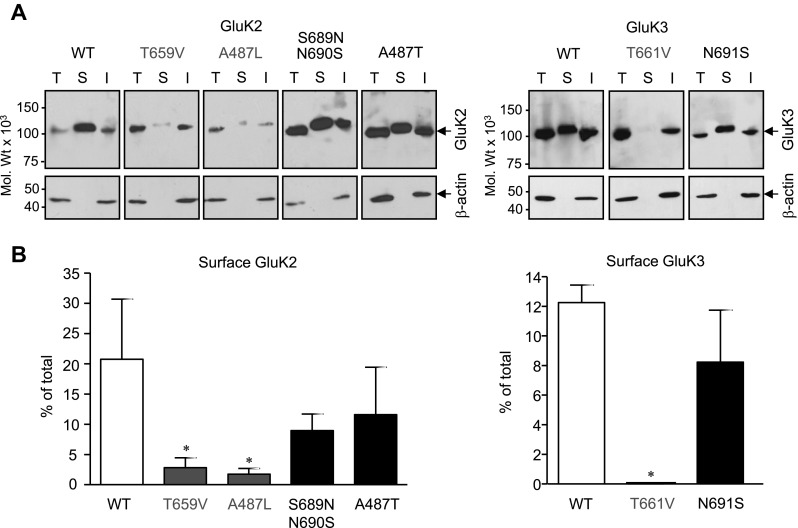
Thr/Val and Ala/Leu mutations reduce cell surface expression of GluK2 and GluK3 subunit proteins. **a** Transiently transfected HEK293 cells expressing wild type (GluK2-WT, GluK3-WT) or mutant subunits with impaired ligand binding sites (GluK2-T659V, GluK2-A487L, GluK3-T661V; red) or conversion mutants with functional ligand binding sites (GluK2-S689N/N690S, GluK2-A487T, GluK3-N691S; blue) were surface biotinylated 24 h after transfection. Following the solubilisation of membranes (total; T), biotin-labelled (cell surface exposed; S) proteins were separated from non-biotinylated (intracellular; I) proteins using streptavidin coated beads. The different subunit populations were analysed using immunoblotting with a GluK2/3 specific antibody. The β-actin content of the samples was analysed to confirm that biotinylation only labelled cell surface exposed proteins in transfected HEK293 cells. Due to differences in total expression levels of various KAR subunit mutants (Fig. 4), immunodetection of bands were optimised for the quantitative comparison of biotinylated and non-biotinylated (intracellular) bands for each construct. **b** Bar diagrams compare the biotinylated (surface) fractions of subunits expressed as % of total. Mutants with impaired LBDs (GluK2-T659V, GluK2-A487L and GluK3-T661V; red bars) show reduced cell surface expression (biotinylation) than the corresponding WT subunits (white bars). In contrast, LBD conversion mutants of GluK2 and GluK3 that retain ligand binding (GluK2-S689N/N690S, GluK2-A487T, GluK3-N691S; blue bars) show no significant change in cell-surface biotinylation compare to WT. Data are mean ± SEM (*n* = 3), *p < 0.05, Student’s *t* test. (Color figure online)

Previous studies reported that LBD mutations that impair glutamate binding or prevent conformational changes can prevent the export of KAR subunit proteins to the cell surface [17, 46]. Therefore, we compared the trafficking of WT and LBD mutant homomeric GluK1–3 KARs by analysing their glycosylation status by EndoH and EndoF digestion (Fig. 5) and cell surface biotinylation (Fig. 6). Our previous studies established that EndoH resistance directly correlates to cell surface expression of KARs and can be used to monitor the processing of glycosylated subunits during their trafficking through the secretory pathway in transfected HEK293 cells and neurons [32, 40]. Therefore, we compared the EndoH resistant (higher molecular weight) KAR subunit protein bands that indicates trafficking through the Golgi, to EndoH sensitive (deglycosylated lower molecular weight) bands that represent ER-retained subunits (Fig. 5). Due to the endoplasmic reticulum retention of WT GluK1 [9], LBD mutants of this subunit (GluK1-T503A, GluK1-T675V and GluK1-N705S/S706N) displayed equally high level of EndoH sensitivity and low level of surface expression with no detectable differences between WT and mutants (Fig. 5b). As expected, ~ 50% of GluK2-WT and ~ 30% of GluK3-WT were EndoH resistant (Fig. 5), which is consistent with previous studies [32, 40]. While the EndoH/EndoF sensitivity profiles of mutant GluK2 and GluK3 with functional LBD (GluK2-S689N/N690S, GluK2-A487T, GluK3-N691S; Fig. 5) were very similar to the corresponding WT, subunits with impaired ligand binding displayed a significantly higher level of EndoH sensitivity (GluK2-T659V, GluK2-A487L, GluK3-T661V; Fig. 5).

Changes in the surface expression of WT and LBD mutant GluK2 and GluK3 homomeric KARs was confirmed by surface biotinylation of transiently transfected HEK293 cells and subsequent separation of biotin-labelled proteins by streptavidin [11, 41]. Total (T), biotin labelled surface (S) and unlabelled intracellular (I) protein fractions were analysed using immunoblotting [32, 40] (Fig. 6a). Cell surface expression levels were determined relative to the total KAR subunit content of the relevant samples (Fig. 6b). No immunoreactivity to β-actin was detected in the biotinylated fractions of the subunits indicating that only proteins present on the cell surface were labelled (Fig. 6a). In agreement with the EndoH sensitivity profiles, there were no significant differences between the surface expression of mutant GluK2 and GluK3 with functional LBD (GluK2-S689N/N690S, GluK2-A487T, GluK3-N691S; Fig. 6b) compared to the corresponding WT (GluK2-WT, GluK3-WT; Fig. 6b). In contrast, the cell surface expression of subunits with impaired LBD was significantly lower (GluK2-T659V, GluK2-A487L, GluK3-T661V; Fig. 6b) compared to the corresponding WT.

These experiments indicate that disruption of ligand binding to GluK2 or GluK3 prevents the cell surface expression of homomeric KARs.

### The Effect of GluK5 Co-expression on the Cell Surface Trafficking of GluK2 LBD Mutants

**Fig. 7:**
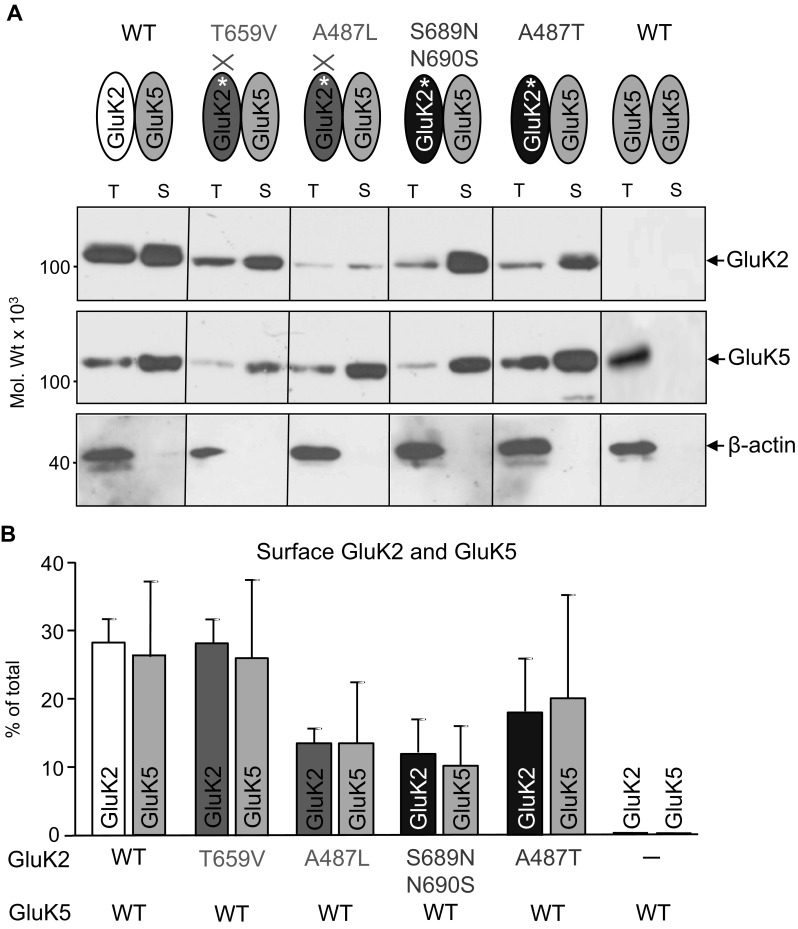
GluK5 co-expression restores the cell surface trafficking of GluK2 with Thr/Val or Ala/Leu mutations. **a** Wild type (WT) and mutant GluK2 subunits (GluK2-T659V, GluK2-A487L, GluK2-S689N/N690S, GluK2-A487T) were co-expressed with the GluK5 WT subunit in transiently transfected HEK293 cells. Following surface biotinylation, labelled proteins (S) were isolated using streptavidin coated beads and analysed using immunoblotting using anti-GluK2/3 and anti-GluK5 antibodies. T is 10% of total protein. The β-actin immunostaining confirmed that biotinylation only labelled cell surface exposed proteins. **b** Comparison of surface (biotinylated) GluK2 and GluK5 subunits (% of total) indicates no significant differences between the various LBD mutant and WT subunits (Data are mean ± SEM, *n* = 3, *p < 0.05, Student’s *t* test). As expected, the intracellularly retained GluK5-WT [11] was not detectable on the cell surface when it was expressed without GluK2

Cell surface expression of heteromeric assemblies of GluK2 with functional (GluK2-WT, GluK2-S689N/N690S, GluK2-A487T) and impaired (GluK2-T659V, GluK2-A487L) LBDs in combination with GluK5-WT was assessed using biotinylation of surface proteins in transiently transfected HEK293 cells (Fig. 7). While GluK5 alone is completely retained in the ER [11], co-expression with WT or any of the LBD mutant GluK2 enabled the surface expression of GluK2/GluK5 heteromers.

Co-expression with GluK5-WT also increased the cell surface expression of all GluK2 variants (GluK2-WT/GluK5-WT: ~ 28%/~ 27%; GluK2-T659V/GluK5-WT: ~ 28%/~ 26%; GluK2-A487L/GluK5-WT: ~ 14%/~ 14%; GluK2-S689N/N690S/GluK5-WT: ~ 12%/~ 10%; GluK2-A487T/GluK5-WT: ~ 18%/~ 20%) compare to homomeric subunits (Fig. 6). This GluK5-WT co-expression-related increase in cell surface GluK2 was particularly prominent for mutant subunits with impaired LBD (~ sevenfold for GluK2-T659V and ~ fivefold for GluK2-A487L; Figs. 6, 7). This indicates that co-expression relieves the ER retention of both GluK5-WT and GluK2 with mutations that block ligand binding (GluK2-T690V, GluK2-A487L) and enables their cell surface trafficking as heteromers.

### Functional Characterisation of Homomeric and Heteromeric KARs

**Fig. 8:**
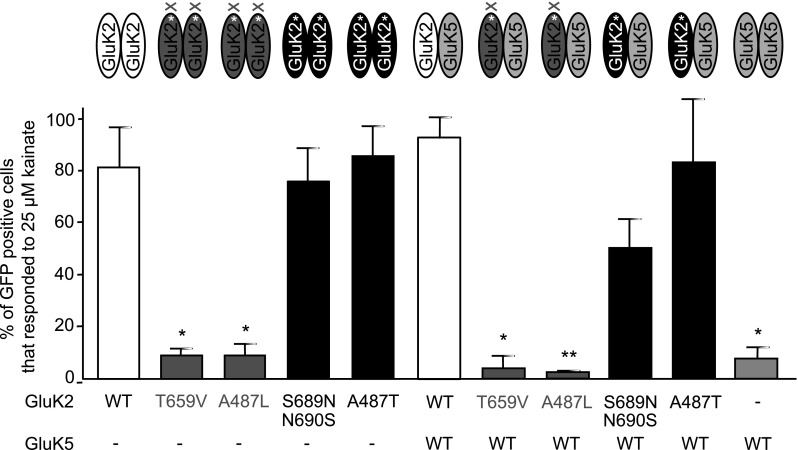
Thr/Val and Ala/Leu mutations of GluK2 abolish kainate-induced responses of both homomeric GluK2 and heteromeric GluK2/GluK5 receptors. Wild type (WT) or mutant (GluK2-T659V, GluK2-A487L, GluK2-S689N/N690S, GluK2-A487T) GluK2 was expressed as homomer or heteromer with WT GluK5 in HEK293 cells as indicated. Following incubation with fura-2-AM, 25 µM kainate-induced increase in [Ca^2+^]_i_ was measured in GFP positive transfected cells. GluK2 mutants with impaired LBD (GluK2-T659V and GluK2-A487L; red bars) have significantly fewer cells that respond to kainate with an increase in [Ca^2+^]_i_ compare to GluK2-WT or subunit conversion mutants (GluK2-S689N/N690S, GluK2-A487T; blue bars). Co-expression of GluK5-WT has not altered the kainate-evoked responses of LBD mutant GluK2 subunits. Data are mean ± SEM (*n* = 3), *p < 0.05, ***p < 0.005 mutants compared to WT, Student’s *t* test. (Color figure online)

Kainate (25 µM) induced changes in intracellular Ca^2+^ concentration ([Ca^2+^]_i_) were analysed using a fluorescence-based assay in transiently transfected HEK293 cells expressing GluK2-WT, GluK2-T659V, GluK2-A487L, GluK2-S689N/N690S or GluK2-A487T either as homomeric receptors or in combination with GluK5-WT (Fig. 8). GFP was used as a marker to identify transfected HEK293 cells (~ 12%) that express KAR subunits. The muscarinic acetylcholine receptor agonist carbachol (10 µM) was used to confirm the responsiveness of transfected cells. Following the investigation of agonist-induced [Ca^2+^]_i_ responses, cells were routinely harvested for immunoblot analysis to verify the expression levels of KAR subunits. Activation (kainate, 25 µM) of mutant GluK2 subunits with functional ligand binding sites (GluK2-S689N/N690S, GluK2-A87T) produced very similar [Ca^2+^]_i_ responses to GluK2-WT, which was not altered significantly by the co-expression of GluK5-WT subunit (Fig. 8). This indicates that exchanging non-conserved residues that convert pharmacological properties of the GluK2 LBD but retain ligand binding [32] (Fig. 3), does not alter receptor function. In contrast, GluK2 mutations that impair the LBD (GluK2-T659V, GluK2-A487L; Fig. 3) abolished kainate (25 µM) evoked [Ca^2+^]_i_ responses compared to GluK2-WT (Fig. 8). Co-expression of GluK5-WT with GluK2-T659V or GluK2-A487L does not rescue receptor function and only very few transfected cells responded to kainate (Fig. 8), despite the presence of functional LBDs in GluK5 and improved cell surface expression of GluK2/GluK5 heteromers (Fig. 7). This suggests that agonist binding to GluK5 alone is not sufficient for the opening of the ion channel and functional GluK2 LBDs are required for the activation of GluK2/GluK5 heteromers. Due to the fast desensitisation rate of GluK3 that cannot be blocked by the addition of ConA [47], we did not include GluK3 in [Ca^2+^]_i_ imaging studies.

## Discussion

**Fig. 9:**
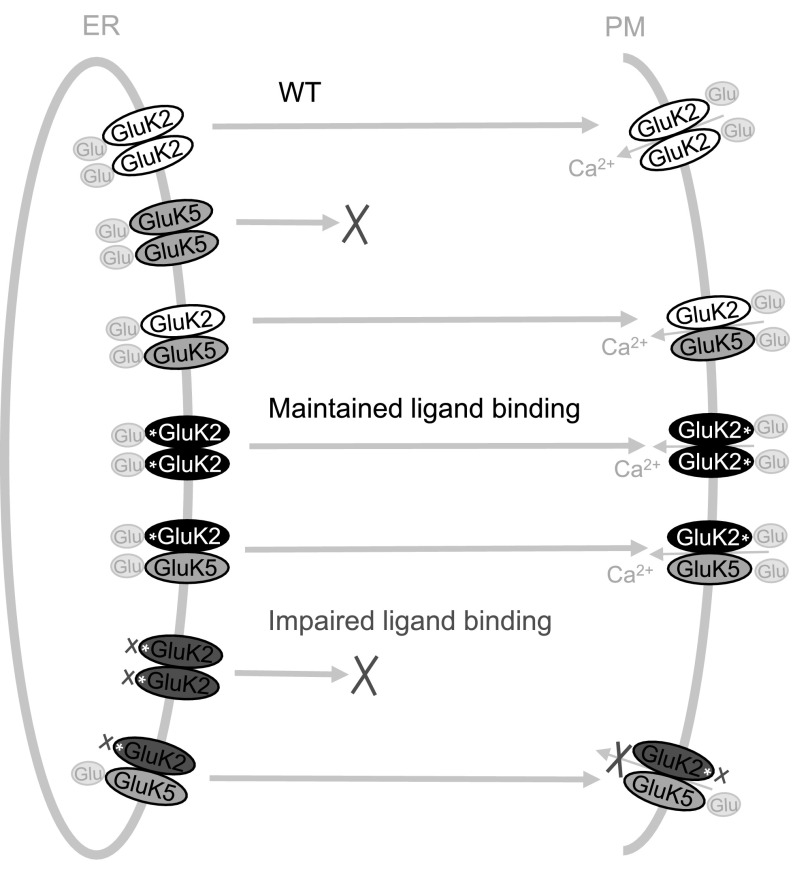
A schematic illustration of the assembly and trafficking of homomeric and heteromeric KARs with functional and impaired ligand binding sites. While GluK2 subunit proteins with functional LBD are readily expressed on the cell surface (*PM* plasma membrane) alone or in combination with GluK5, GluK2 with impaired ligand binding sites are retained in intracellular compartments (*ER* endoplasmic reticulum). The cell surface expression of GluK5 depends on co-assembly with GluK2. Co-expression with GluK5 restores the surface expression of GluK2 subunits with impaired LBD (red), but these receptors are non-functional despite the presence of active ligand binding sites in GluK5. This suggests that agonist binding to GluK5 is not sufficient to open the ion channel and activation of GluK2 is required for the activation of GluK2/GluK5 heteromeric KARs. (Color figure online)

We found that mutations of conserved residues in the LBDs (GluK2-A487, GluK1-T675, GluK2-T659, GluK3-T661) hindered both kainate and MG binding to all low affinity subunits of KARs. In contrast, exchanging non-conserved T/A amino acid residues between GluK1 and GluK2 subunits (GluK1-T503A, GluK2-A487T, GluK3-T489A) did not alter kainate binding. While reciprocal mutations of the non-conserved N/S residues in GluK1–3 (GluK1-N705S/S706N, GluK2-S689N/N690S, GluK3-N691S) enabled agonist binding, the kainate binding activities of mutant subunits shifted from the parent subunit towards that of the subunit the mutation was designed to imitate. In line with previous studies, mutations that impair ligand binding to GluK2 and GluK3 also disrupted the trafficking of homomeric KARs and lead to the intracellular retention of these subunit proteins [17, 46] (Fig. 9). In contrast, exchanging non-conserved residues in the GluK2 and GluK3 LBDs has no significant effect on their glycosylation and cell surface expression profiles. Co-assembly with the WT high affinity GluK5 subunit can override ER retention of mutant GluK2 with impaired LBD and promotes the cell surface expression of both subunits. However, [Ca^2+^]_i_ imaging indicates that the occupancy of both GluK2 and GluK5 LBDs is required for the full activation of heteromeric KAR channels (Fig. 9).

Previous studies revealed that ligand binding and associated conformational changes are necessary for the biogenesis of KARs and their trafficking from the ER through the Golgi network to the cell surface in both homo- and heteromeric assemblies [14–16, 46, 48]. Our study of conserved and non-conserved residues in the LBDs of GluK1–3 with impaired and maintained ligand binding activities confirmed and extended these studies to other KAR subunits and amino acid residues. While the occupancy of the LBD enhances the cell surface expression of KARs, the opening of the ion channel pore appears to be not necessary [14, 46]. However, restricting the ligand-induced conformational changes reduces the cell surface expression of KARs [15, 18, 46]. The intracellular retention is not due to gross protein misfolding, because LBD mutant KAR subunits are capable of assembly [14, 15] and do not show increased degradation rate [15]. Our results are consistent with these findings. When expressed individually, GluK2 subunits with impaired LBDs (GluK2-A487L, GluK2-T659V) and GluK5-WT are all retained in the ER with very limited or no cell surface trafficking and no detectable change in degradation rates. However, when GluK2-A487L or GluK2-T659V was co-expressed with GluK5-WT, the ER retention domains of GluK5 [11, 12, 49] was shielded in the GluK2/GluK5 heteromer and enabled the surface expression of both subunits. This also suggests that glutamate binding to GluK5 alone is sufficient for the cell surface expression of the GluK2/GluK5 complex. Indeed, the disruption of glutamate binding to GluK5 leads to a reduction in the plasma membrane expression and intracellular retention of GluK2/GluK5 heteromers [15, 16]. Collectively these findings suggest a dominant role for the GluK5 LBD in the glutamate mediated cell surface trafficking of the GluK2/GluK5 complex. The GluK5 subunit may act as a possible modulator of GluK2 trafficking or folding, ensuring that the protein reaches the correct conformation to be trafficked.

GluK2 and GluK5 are both widely expressed in the CNS [50] and they preferentially assemble [42] with 2:2 stoichiometry [7] as pairs of heterodimers [51, 52] to form functional KARs. Unlike GluK2, GluK5 does not form functional homomeric channels even when the ER retention/retrieval motif is removed [15]. Because functional expression of GluK5 requires co-assembly with other KARs subunits, it is difficult to investigate the pharmacological properties of GluK5 in isolation. Newly synthesised KAR antagonists are routinely tested on subunits expressed in HEK293 cells for their ability and selectivity to reduce l-glutamate induced calcium-influx by using a FlexStation [6, 33]. Based on previous studies we anticipated that the co-expression of GluK5-WT with impaired LBD containing GluK2 would provide a cell-based assay for the screening of GluK5 selective ligands. While the presence of the functional LBD in GluK5 and the masking of ER retention motifs in GluK5 by GluK2 LBD mutants enable the cell surface trafficking of GluK5-WT/GluK2-T659V and GluK5-WT/GluK2-A487L heteromers, exposure to kainate does not produce changes in [Ca^2+^]_i_. This suggests that occupancy of GluK5 LBDs in the heteromeric assembly is sufficient for forward trafficking of the subunit assembly, but agonist binding to GluK2 is required for the opening of the KAR channel pore. Furthermore, previous pharmacological studies of GluK1/GluK5 heteromeric complexes demonstrated that GluK1 can gate current without concomitant activation of their partner GluK5 subunits and occupancy of binding sites on individual KAR subunits activates distinct channel conductance and kinetic properties [53]. It is also possible to activate GluK2/GluK5 heteromers through agonist binding to the GluK2 subunit while GluK5 is inhibited by domoate [36, 54]. Even though the individual subunits can be activated separately, the GluK2 subunit may still need to be bound to a ligand and inducing a prolonged current before the GluK5 can react to bound agonist. Another possible explanation for the lack of activation seen in heteromeric assemblies is that when there is no ligand bound to the GluK2 subunit only the GluK5 subunit is able to react. It is not known if ConA effectively blocks the desensitisation of GluK5, which could mean that the GluK5 subunit is desensitising too quickly to produce a detectable change in [Ca^2+^]_i_. However, electrophysiological studies suggest that activation of GluK5 subunits by agonist activates the KARs without inducing desensitisation [36, 55]. Another possibility is that the LBD dimer interface in the GluK5-WT/GluK2-T659V and GluK5-WT/GluK2-A487L hetero-dimers is not stable when the GluK5 LBD is closed, and the GluK2 LBD is open, which may promote entry into the desensitised state and/or enhance the rate of deactivation. Stability of the dimer interface and of the hinge region, which serves as the pivot point for LBD closure, has been shown to be necessary to block desensitisation and slow deactivation, respectively, of AMPARs [56, 57]. While it is not possible to perform functional assays, it may be possible to use the GluK5-WT/GluK2-T659V and GluK5-WT/GluK2-A487L heteromers in competition binding assays to screen compounds on GluK5.

The biosynthesis, assembly and cell surface trafficking of KARs are key determinants of neuronal network excitability in the CNS [1, 3]. This study further highlights the pharmacological chaperone role of glutamate in KAR trafficking and the differential functions of various subunits. Defining the distinct roles of various subunits is essential for the development of selective ligands for the targeted modulation of KARs in the CNS. Intriguingly, epilepsy-associated mutations in the GluN2A NMDAR subunit that caused reduction in agonist potency also showed significantly decreased expression levels and trafficking to the cell surface [58]. Functional and pharmacological analyses of mutant KAR and other iGluR subunits [47, 58–60] would provide interesting insight into the molecular mechanism of associated neurological and psychiatric disorders.

## Abbreviations

AMPA: (*S*)-2-Amino-3-hydroxy-5-methyl-4-isoxazolepropionic acid
[Ca^2+^]_i_: Intracellular Ca^2+^ concentration
ConA: Concanavalin A
ECL: Enzyme chemiluminescence
EndoF: Peptide-*N*-glycosidase F
EndoH: Endoglycosidase H
ER: Endoplasmic reticulum
GABA: γ-Aminobutyric acid
GFP: Green fluorescent protein
GluA1-4: AMPA receptor subunit 1–4
GluK1–5: According to the new IUPHAR nomenclature the KAR subunits that were formerly known as GluR5–7 (or GLUK5–7) are now named GluK1–3 and those previously called KA-1 and KA-2 (or GLUK1 and GLUK2) are now named GluK4 and GluK5.
[^3^H]MG: [^3^H]-(2*S*,4*R*)-4-methylgutamate
iGluR: Ionotropic glutamate receptor
HEK: Human embryonic kidney
KA: Kainate, (2*S*,3*S*,4*S*)-3-carboxymethyl-4-isopropenyl-pyrrolidine-2-carboxylic acid
KAR: Kainate receptor
LBD: Ligand binding domain
NMDA: *N*-Methyl-d-aspartate
WT: Wild type

## References

[1] Lerma (2006). Kainate receptor physiology. Curr Opin Pharmacol.

[2] Molnár E, Choi S (2018). Glutamate receptors. Encyclopedia of signaling molecules.

[3] Lerma J, Marques JM (2013). Kainate receptors in health and disease. Neuron.

[4] Valbuena S, Lerma J (2016). Non-canonical signaling, the hidden life of ligand-gated ion channels. Neuron.

[5] Møllerud S, Frydenvang K, Pickering DS, Kastrup JS (2017). Lessons from crystal structures of kainate receptors. Neuropharmacology.

[6] Jane DE, Lodge D, Collingridge GL (2009). Kainate receptors: pharmacology, function and therapeutic potential. Neuropharmacology.

[7] Reiner A, Arant RJ, Isacoff EY (2012). Assembly stochiometry of the GluK2/GluK5 kainate receptor complex. Cell Rep.

[8] Bernard A, Ferhat L, Dessi F, Charton G, Represa A, Ben-Ari Y, Khrestchatisky M (1999). Q/R editing of the GluR5 and GluR6 kainate receptors in vivo and in vitro: evidence for independent developmental, pathological and cellular regulation. Eur J Neurosci.

[9] Pahl S, Tapken D, Haering SC, Hollmann M (2014). Trafficking of kainate receptors. Membranes.

[10] Evans AJ, Gurung S, Henley JM, Nakamura Y, Wilkinson KA (2018). Exciting times: new advances towards understanding the regulation and roles of kainate receptors. Neurochem Res.

[11] Gallyas F, Ball S, Molnar E (2003). Assembly and cell surface expression of KA-2 subunit-containing kainate receptors. J Neurochem.

[12] Ren Z, Riley NJ, Garcia EP, Sanders JM, Swanson GT, Marshall J (2003). Multiple trafficking signals regulate kainate receptor KA2 subunit surface expression. J Neurosci.

[13] Nasu-Nishimura Y, Hurtado D, Braud S, Tang TTT, Isaac JTR, Roche KW (2006). Identification of an endoplasmic reticulum-retention motif in an intracellular loop of the kainate receptor subunit KA2. J Neurosci.

[14] Mah SJ, Cornell E, Mitchell NA, Fleck MW (2005). Glutamate receptor trafficking: endoplasmic reticulum quality control involves ligand binding and receptor function. J Neurosci.

[15] Valluru L, Xu J, Zhu Y, Yan S, Contractor A, Swanson GT (2005). Ligand binding is a critical requirement for plasma membrane expression of heteromeric kainate receptors. J Biol Chem.

[16] Fisher JL, Housley PR (2013). Agonist binding to the GluK5 subunit is sufficient for functional surface expression of heteromeric GluK2/GluK5 kainate receptors. Cell Mol Neurobiol.

[17] Fleck M (2006). Glutamate receptors and endoplasmic reticulum quality control: looking beneath the surface. Neuroscientist.

[18] Priel A, Selak S, Lerma J, Stern-Bach Y (2006). Block of kainate receptor desensitisation uncovers a key trafficking checkpoint. Neuron.

[19] Sander T, Hildmann T, Kretz R, Furst R, Sailer U, Bauer G, Schmitz B, Beck-Mannagetta G, Wienker TF, Janz D (1997). Allelic association of juvenile absence epilepsy with a GluR5 kainate receptor gene (GRK1) polymorphism. Am J Med Genet.

[20] Li JM, Zeng YJ, Peng F, Li L, Yang TH, Hong Z, Lei D, Chen Z, Zhou D (2010). Aberrant glutamate receptor 5 expression in temporal lobe epilepsy lesions. Brain Res.

[21] Crépel V, Mulle C (2015). Physiopathology of kainate receptors in epilepsy. Curr Opin Pharmacol.

[22] Casey JP (2012). A novel approach of homozygous haplotype sharing identifies candidate genes in autism spectrum disorder. Hum Genet.

[23] Aller MI, Pecoraro V, Paternain AV, Canals S, Lerma J (2015). Increased dosage of high-affinity kainate receptor gene grik4 alters synaptic transmission and reproduces autism spectrum disorders features. J Neurosci.

[24] Motazacker MM, Rost BR, Hucho T, Garshasbi M, Kahrizi K, Ullmann R (2007). A defect in the ionotropic glutamate receptor 6 gene (GRIK2) is associated with autosomal recessive mental retardation. Am J Hum Genet.

[25] Formicola D, Aloia A, Sampaolo S, Farina O, Diodato D, Griffiths LR, Gianfrancesco F, Di Iorio G, Esposito T (2010). Common variants in the regulative regions of GRIA1 and GRIA3 receptor genes are associated with migraine susceptibility. BMC Med Genet.

[26] Pickard BS, Malloy MP, Christoforou A, Thomson PA, Evans KL, Morris SW (2006). Cytogenetic and genetic evidence supports a role for the kainate-type glutamate receptor gene, GRIK4, in schizophrenia and bipolar disorder. Mol Psychiatry.

[27] Luciano M, Houlihan LM, Harris SE, Gow AJ, Hayward C, Starr JM, Deary IJ (2010). Association of existing and new candidate genes for anxiety, depression and personality traits in older people. Behav Genet.

[28] Knight HM, Walker R, James R, Porteous DJ, Muir WJ, Blackwood DH, Pickard BS (2012). GRIK4/KA1 protein expression in human brain and correlation with bipolar disorder risk variant status. Am J Med Genet.

[29] Falcón-Moya R, Sihra T, Rodríguez-Moreno A (2018). Kainate receptors: role in epilepsy. Front Mol Neurosci.

[30] Zhuo M (2017). Ionotropic glutamate receptors contribute to pain transmission and chronic pain. Neuropharmacology.

[31] Zhou M (2017). Cortical kainate receptors and behavioural anxiety. Mol Brain.

[32] Atlason PT, Scholefield CL, Eaves RJ, Mayo-Martin MB, Jane DE, Molnár E (2010). Mapping the ligand binding sites of kainate receptors: molecular determinants of subunit-selective binding of the antagonist [^3^H]UBP310. Mol Pharmacol.

[33] Larsen AM, Bunch L (2011). Medicinal chemistry of competitive kainate receptor antagonists. ACS Chem Neurosci.

[34] Kaczor AA, Karczmarzyk Z, Fruzin A, Pihlaja K, Sinkkonen J, Wiinamaki K, Kronbach C, Unverferth K, Poso A, Matosiuk D (2014). Structural studies, homology modelling and molecular docking of novel non-competitive antagonists of GluK1/GluK2 receptors. Bioorg Med Chem.

[35] Larsen AP, Fièvre S, Frydenvang K, Francotte P, Pirotte B, Kastrup JS, Mulle C (2017). Identification and structure-function study of positive allosteric modulators of kainate receptors. Mol Pharmacol.

[36] Fisher MT, Fisher JL (2014). Contributions of different kainate receptor subunits to the properties of recombinant homomeric and heteromeric receptors. Neuroscience.

[37] Solly K, Klein R, Rudd M, Holloway MK, Johnson EN, Henze D, Finley MFA (2015). High-throughput screen of GluK1 receptor indentifies selective inhibitors with a variety of kinetic profiles using fluorescence and electrophysiology assays. J Biomol Screen.

[38] Perrais D, Pinheiro PS, Jane DE, Mulle C (2009). Antagonism of recombinant and native GluK3-containing kainate receptors. Neuropharmacology.

[39] Mayer ML (2005). Crystal structures of the GluR5 and GluR6 ligand binding cores: molecular mechanisms underlying kainate receptor selectivity. Neuron.

[40] Ball SM, Atlason PT, Shitu-Balogun OO, Molnár E (2010). Assembly and intracellular distribution of kainate receptors is determined by RNA editing and subunit composition. J Neurochem.

[41] Molnár E, Luján R, Ciruela F (2016). Investigation of neurotransmitter receptors in brain slices using cell surface biotinylation. Receptor and ion channel detection in the brain. Neuromethods.

[42] Wenthold RJ, Trumpy VA, Zhu WS, Petralia RS (1994). Biochemical and assembly properties of GluR6 and KA2, two members of the kainate receptor family, determined with subunit specific antibodies. J Biol Chem.

[43] Molnar E, Varadi A, McIlhinney RAJ, Ashcroft SJH (1995). Identification of functional ionotropic glutamate receptor proteins in pancreatic β-cells and in islets of langerhans. FEBS Lett.

[44] Gladding CM, Collett VJ, Jia Z, Bashir ZI, Collingridge GL, Molnár E (2009). Tyrosine dephosphorylation regulates AMPAR internalisation in mGluR-LTD. Mol Cell Neurosci.

[45] Mott DD, Rojas A, Fisher JL, Dingledine RJ, Benveniste M (2010). Subunit-specific desensitization of heteromeric kainate receptors. J Physiol.

[46] Gill MB, Vivithanaporn P, Swanson GT (2009). Glutamate binding and conformational flexibility of ligand-binding domains are critical early determinants of efficient kainate receptor biogenesis. J Biol Chem.

[47] Matute C (2011). Therapeutic potential of kainate receptors. CNS Neurosci Ther.

[48] Fleck MW, Cornell E, Mah SJ (2003). Amino-acid residues involved in glutamate receptor 6 kainate receptor gating and desensitization. J Neurosci.

[49] Hayes DM, Braud S, Hurtado DE, McCallum J, Standley S, Isaac JT, Roche KW (2003). Trafficking and surface expression of the glutamate receptor subunit, KA2. Biochem Biophys Res Commun.

[50] Petralia RS, Wang YX, Wenthold RJ (1994). Histological and ultrastructural localization of the kainate receptor subunits, KA2 and GluR6/7, in the rat nervous system using selective antipeptide antibodies. J Comp Neurol.

[51] Kumar J, Schuck P, Mayer ML (2011). Structure and assembly mechanism for heteromeric kainate receptors. Neuron.

[52] Paramo T, Brown PMG, Musgaard M, Bowie D, Biggin PC (2017). Functional validation of heteromeric kainate receptor models. Biophys J.

[53] Swanson GT, Green T, Sakai R, Contractor A, Che W, Kamiya H, Heinemann SF (2002). Differential activation of individual subunits in heteromeric kainate receptors. Neuron.

[54] Fisher JL (2014). The neurotoxin domoate causes long-lasting inhibition of the kainate receptors GluK5 subunit. Neuropharmacology.

[55] Fisher JL, Mott DD (2011). Distinct functional roles of subunits within the heteromeric kainate receptor. J Neurosci.

[56] Sun Y, Olson R, Horning M, Armstrong N, Mayer M, Gouaux E (2002). Mechanism of glutamate receptor desensitisation. Nature.

[57] Jin R, Clark S, Weeks AM, Dudman JT, Gouaux E, Partin KM (2005). Mechanism of positive allosteric modulators acting on AMPA receptors. J Neurosci.

[58] Addis L, Virdee JK, Vidler LR, Collier DA, Pal DK, Ursu D (2017). Epilepsy-associated *GRIN2A* mutations reduce NMDA receptor trafficking and agonist potency—molecular profiling and functional rescue. Sci Rep.

[59] Yuan H, Low C-M, Moody OA, Jenkins A, Traynelis SF (2015). Ionotropic GABA and glutamate receptor mutations and human neurologic diseases. Mol Pharmacol.

[60] Xu XX, Luo JH (2018). Mutations of *N*-methyl-D-aspartate receptor subunits in epilepsy. Neurosci Bull.

